# Dissolution of Platinum: Limits for the Deployment of Electrochemical Energy Conversion?**

**DOI:** 10.1002/anie.201207256

**Published:** 2012-11-04

**Authors:** Angel A Topalov, Ioannis Katsounaros, Michael Auinger, Serhiy Cherevko, Josef C Meier, Sebastian O Klemm, Karl J J Mayrhofer

**Affiliations:** Department of Interface Chemistry and Surface Engineering, Max-Planck-Institut für Eisenforschung GmbHMax-Planck-Strasse 1, 40237 Düsseldorf (Germany) E-mail: topalov@mpie.demayrhofer@mpie.de Homepage: http://www.mpie.de/ecat; Center for Electrochemical Sciences, Ruhr-Universität Bochum, Universitätsstrasse 15044780 Bochum (Germany)

**Keywords:** catalyst stability, dissolution processes, electrochemistry, fuel cells, platinum

The large-scale conversion of electrical into chemical energy and vice versa, in combination with the utilization of renewable primary energy resources, is considered to be of crucial importance in a sustainable energy concept. Electrochemical energy conversion systems, such as electrolyzers and fuel cells, are promising candidates for that purpose; however, they yet suffer from various shortcomings that impede their cost-effective deployment. In particular, the transition from fossil-fuel-based combustion engines to low-temperature fuel cells, which will be a major milestone for automotive applications, is mainly limited by the performance and the cost of the electrocatalyst materials.^1^ While a lot of progress has been recently made in the development of low-cost, non-noble electrocatalysts,^2^ they continue to lag in performance compared to the state-of-the-art platinum-based catalyst materials.^3^ Regarding the latter, major research efforts over the last decades have also led to an improved understanding of the electrochemical reactions and to the design of more active catalysts, especially for the oxygen reduction by alloying Pt with transition metals, which at the same time decreases the required amount of the precious metal.^4^ However, the long-term stability of Pt and Pt-alloy electrocatalysts as requested by the US Department of Energy (DoE) fuel-cell targets still remains a challenge.^5^

Mitigation of catalyst degradation by more directed design strategies requires a thorough understanding of the underlying principles. Significant insights into the nanoscale phenomena occurring on finely dispersed supported Pt catalysts have already been achieved, for instance by degradation studies combining standard electrochemical techniques and electron microscopy.^6^ Degradation mechanisms, such as particle agglomeration, detachment, or support corrosion that may occur on such catalysts are closely related to the structure, morphology, and composition of the high-surface-area catalysts. Platinum dissolution, however, is a fundamental process that can be also studied effectively on extended surfaces, but it has been still ambiguously discussed in the literature. For example, it is not clear, even for polycrystalline Pt, whether Pt dissolves anodically as a competition to oxide formation, cathodically during oxide reduction, or by chemical dissolution of the formed oxide.^7^ Furthermore, the correlations of the operation conditions with the nature and extent of dissolution have not been elucidated sufficiently. In this initial study, we investigate and quantify the dissolution of polycrystalline Pt in acidic media at room temperature for different operating parameters, under both potentiostatic and potentiodynamic conditions, by utilizing an electrochemical scanning flow cell (SFC) directly coupled to an inductively coupled plasma mass spectrometer (ICP-MS). This unique experimental technique enables the investigation of stability of electrode materials by highly sensitive online elemental analysis of the electrolyte in parallel to conventional electrochemical measurements.^8^

Even though a fuel cell typically operates under almost steady-state conditions below 1.0 V, the catalyst on the cathode side can locally be exposed to potentials up to 1.5 V during startup or shutdown of the cell.^9^ To understand the impact of the anodic polarization on the amount of dissolved Pt during potentiodynamic conditions, potential cycling was performed using different positive potential limits (Figure 1). During potential cycling within only the hydrogen adsorption and double-layer region, the concentration of Pt in the electrolyte remains below the detection limit of the ICP-MS (10 ppt, corresponding to ca. 3 pg cm^−2^ s^−1^). Interestingly, there is no detectable increase in the amount of dissolved Pt even when potential cycling is performed up to the region of adsorption/desorption of oxygenated species on/from Pt. Only when the potential cycling exceeds ca. +1.15 V_RHE_, Pt starts to dissolve considerably so that it can be detected downstream (Figure 1 b). With a further increase of the upper vertex potential, the amount of dissolved Pt becomes more pronounced and two distinct peaks start to evolve in the dissolution profile (Figure 1 c). One peak appears parallel to the oxidation of the Pt surface in the positive-going scan and is rather independent of the upper vertex potential. The second, more dominating peak occurs during the reduction of the surface in the negative-going scan, starting below about +1.0 V_RHE_, and depends strongly on the positive potential limit. This can be more clearly seen in Figure 1 e, where the measured Pt concentration is plotted versus the applied potential for representative cyclic voltammograms (CVs). The two peaks can be correlated to the electrode processes occurring concomitantly, namely the electrooxidation of Pt and the electroreduction of the oxidized surface. While the minor oxidative process can be rationalized (electro)chemically by direct dissolution of Pt or its oxide, the dissolution of Pt in the negative-going sweep below the Pt^2+^/Pt standard potential seems to be more complex. Although the exact nature of the oxide species on Pt is yet still unresolved, it has been proposed that the oxidation of the surface atoms is complete above about +1.15 V_RHE_, and that a further increase in the potential leads to the formation of a so-called sub-surface oxide.^10^ As a significant increase in the dissolved amount of Pt is observed during the negative-going sweep when the upper potential limit is exceeding +1.15 V_RHE_, it is very likely that the observed reductive dissolution is related to the presence of the sub-surface oxide. The latter weakens the Pt–Pt bonds, and upon the reduction of the surface and subsurface oxide, the outermost Pt can detach, triggering significant Pt dissolution.

**Figure 1:**
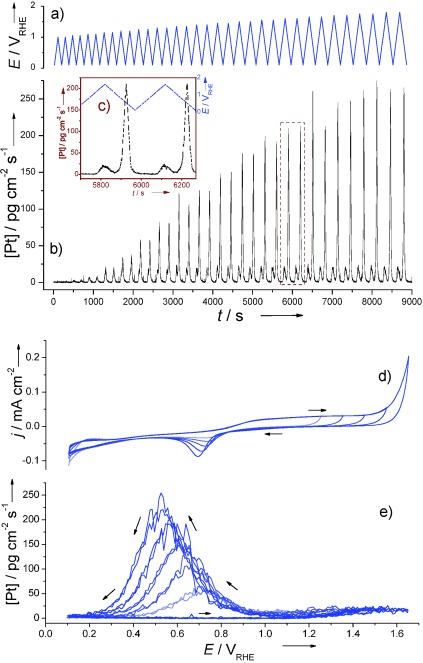
a) The applied experimental sequence in 0.1 m HClO_4_, consisting of two cyclic voltammograms with a scan rate of 0.01 V s^−1^ for each potential window that always start from +0.1 V_RHE_ to an upper potential limit between +1.0 and +1.8 V_RHE_ raised in steps of 0.05 V_RHE_. b) Corresponding time-resolved dissolution profile of Pt presented on the same time axis as in (a). c) A magnification on the region around 6000 s. d) Representative cyclic voltammograms with e) their corresponding mass-spectrometric voltammograms.

The amount of dissolved Pt for each peak can be quantified by the integration of the instant Pt concentration in the electrolyte over time, taking into account the rate of the electrolyte flow.8a The sum of the two integrals for the oxidative and the reductive peak yields the total amount of Pt that dissolves during a single cycle. The area-normalized amount of Pt per cycle (Figure 2 a) increases monotonically with the upper potential limit, namely from non-detectable amount at potentials below +1.05 V_RHE_ to almost 10 ng cm^−2^ cycle^−1^ at +1.8 V_RHE_. The latter corresponds to dissolution of about 2.4 % of a monolayer per cycle, considering a surface density of Pt atoms of 1.3×10^15^ cm^−2^. Although this is rather negligible for bulk Pt, such a dissolution rate, if proportional to surface area, could be highly detrimental to high-surface-area nanoparticle catalysts traditionally used in fuel cells. The amount of dissolved Pt is also highly dependent on the duration of a potential cycle; that is, on the scan rate (Figure 2 b). Scan rates below 0.01 V s^−1^ in the potential window from +0.1 to +1.5 V_RHE_ lead to a dissolution of more than 7 ng cm^−2^ cycle^−1^, whereas at scan rates above 0.1 V s^−1^, less than 2 ng cm^−2^ cycle^−1^ is dissolved for the same upper potential limit. It can be concluded from Figure 2 a and b, that during the start/stop of a fuel cell, the potential on the electrode should ideally not exceed +1.1 V_RHE_; if this still occasionally happens, then the potential changes should be performed as fast as possible to reduce the amount of dissolved Pt.

**Figure 2:**
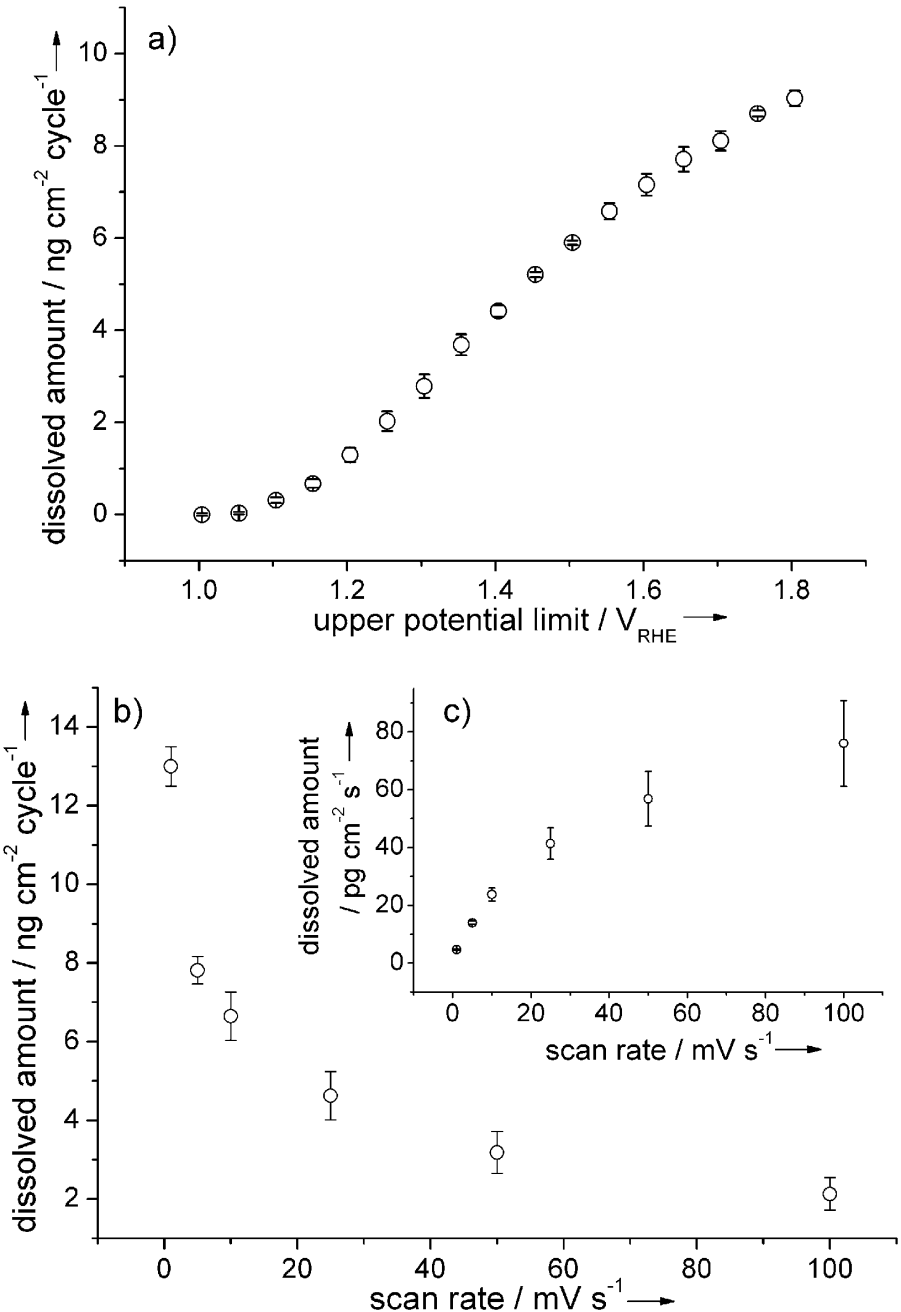
The amount of dissolved Pt normalized per cycle plotted against a) the upper vertex potential for cyclic voltammograms starting from +0.1 V_RHE_ with a scan rate of 0.01 V s^−1^; b) the scan rate for voltammograms from +0.1 to +1.5 V_RHE_; and c) the amount of dissolved Pt normalized per time versus scan rate. The error bars are each based on three independent measurements.

The picture, however, turns around when the dissolution normalized to experiment time is considered, where low scan rates of 0.001 V s^−1^ induce dissolution of only 5 pg cm^−2^ s^−1^, while higher scan rates of 0.1 V s^−1^ lead to dissolution of 76 pg cm^−2^ s^−1^ (Figure 2 c). In the limiting case of no potential variation over time, the Pt dissolution rate becomes negligible. Indeed, in a potential step experiment the rate of Pt dissolution always declines significantly for the first 200 s following a potential perturbation that involves a change in the surface state (Figure 3). The dissolution rate falls below the detection limit even in the critical region of Pt surface oxidation in between +0.8 and +1.3 V_RHE_.

**Figure 3:**
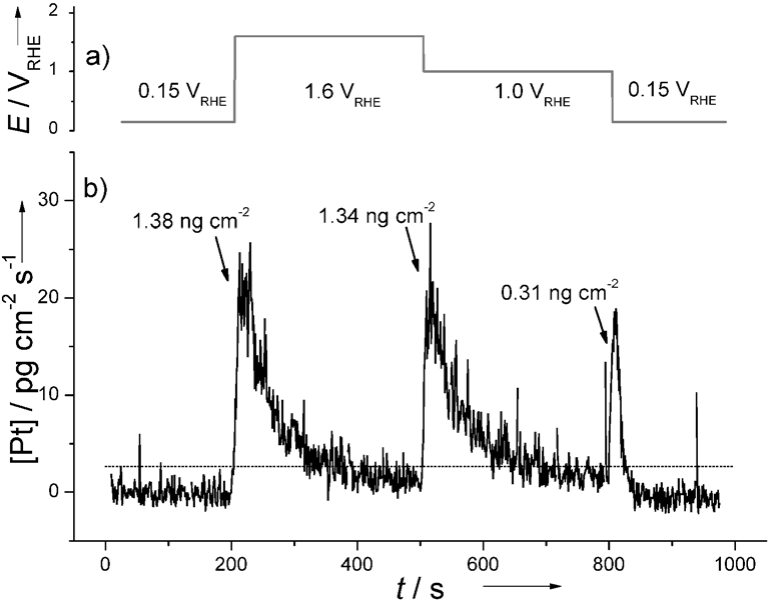
Representative chronoamperometric experiment sequence that demonstrates the typical behavior of Pt dissolution under steady-state conditions. The graph shows a) the applied potential sequence (potential holds at +0.15, +1.6, +1.0, and +0.15 V_RHE_) with b) the corresponding dissolution profile. The dotted line indicates the detection limit for the measurement, as determined from the ICP-MS calibration.

In contrast to other elements that show an active dissolution behavior (for example, copper8a), Pt dissolution is a transient process occurring only when the potential changes cause a substantial change in the surface state. Thus, the dissolution of Pt cannot be explained by reaction equilibria between the oxidized surface, the reduced surface, and dissolved species, which would predict certain constant dissolution rates for different oxide coverage; that is, different applied potentials.^11^ As a consequence, traditional theories also for nanoparticle catalysts will eventually have to be refined to account for the time-dependent Pt dissolution at constant potential. To completely elucidate the dissolution mechanism, extended efforts will be necessary to characterize the exact nature of the surface oxides, the valence of the dissolved species, and the influence of temperature, pH, and mass-transport on the involved reactions. Furthermore, further insights into the degradation of real high-surface-area catalysts need to be provided by the extension of the SFC/ICP-MS setup for the analysis of porous materials.

Independent of the determination of the mechanism, the quantification of Pt dissolution as a function of the experimental parameters presented herein offers highly valuable guidelines for designing and operating Pt-based materials to optimize their durability in fuel cells. The intrinsic properties of polycrystalline Pt alone already indicate that more than mere material developments will be required for achieving all of the desired performance targets, particularly regarding the severe start/stop cycling. Only a combined engineering, electrochemistry, and materials science approach considering the complex interplay of individual components and operation modes within fuel cells will manage to improve the essential durability of electrocatalysts.

## Supplementary material

Detailed facts of importance to specialist readers are published as ”Supporting Information”. Such documents are peer-reviewed, but not copy-edited or typeset. They are made available as submitted by the authors.

## References

[1] Vesborg PCK, Jaramillo TF (2012). RSC Adv.

[2a] Wu G, More KL, Johnston CM, Zelenay P (2011). Science.

[2b] Lefèvre M, Proietti E, Jaouen F, Dodelet J-P (2009). Science.

[3] Gasteiger HA, Markovic NM (2009). Science.

[4a] Srivastava R, Mani P, Hahn N, Strasser P (2007). Angew. Chem.

[b14] (2007). Angew. Chem. Int. Ed.

[4b] Sasaki K, Naohara H, Cai Y, Choi YM, Liu P, Vukmirovic MB, Wang JX, Adzic RR (2010). Angew. Chem.

[b16] (2010). Angew. Chem. Int. Ed.

[4c] Stephens IEL, Bondarenko AS, Grønbjerg U, Rossmeisl J, Chorkendorff I (2012). Energy Environ. Sci.

[5a] http://www.eere.energy.gov/hydrogenandfuelcells/mypp/.

[5b] Mayrhofer KJJ, Arenz M (2009). Nat. Chem.

[6a] Carlton CE, Chen S, Ferreira PJ, Allard LF, Shao-Horn Y (2012). J. Phys. Chem. Lett.

[6b] Meier JC, Katsounaros I, Galeano C, Bongard H, Topalov AA, Kostka A, Karschin A, Schüth F, Mayrhofer KJJ (2012). Energy Environ. Sci.

[6c] Meier JC, Galeano C, Katsounaros I, Topalov AA, Kostka A, Schüth F, Mayrhofer KJJ (2012). ACS Catal.

[6d] Oezaslan M, Heggen M, Strasser P (2012). J. Am. Chem. Soc.

[7a] Johnson DC, Napp DT, Bruckenstein S (1970). Electrochim. Acta.

[7b] Mitsushima S, Koizumi Y, Uzuka S, Ota K-I (2008). Electrochim. Acta.

[7c] Yadav AP, Nishikata A, Tsuru T (2009). J. Electrochem. Soc.

[7d] Inzelt G, Berkes B, Kriston Á (2010). Electrochim. Acta.

[7e] Rinaldo SG, Stumper J, Eikerling M (2010). J. Phys. Chem. C.

[8a] Klemm SO, Topalov AA, Laska CA, Mayrhofer KJJ (2011). Electrochem. Commun.

[8b] Klemm SO, Karschin A, Schuppert AK, Topalov AA, Mingers AM, Katsounaros I, Mayrhofer KJJ (2012). J. Electroanal. Chem.

[9] Rabis A, Rodriguez P, Schmidt TJ (2012). ACS Catal.

[10a] Nagy Z, You H (2002). Electrochim. Acta.

[10b] Jerkiewicz G, Vatankhah G, Lessard J, Soriaga MP, Park Y-S (2004). Electrochim. Acta.

[11] Darling RM, Meyers JP (2003). J. Electrochem. Soc.

[12] Schuppert AK, Topalov AA, Katsounaros I, Klemm SO, Mayrhofer KJJ (2012). J. Electrochem. Soc.

