# Supplementary material for: Dissolution of Platinum: Limits for the Deployment of Electrochemical Energy Conversion?
Source: Angew Chem Int Ed Engl. 2012 Nov 4;51(50):12613–5. doi: 10.1002/anie.201207256 (PMC3556695; doi:10.1002/anie.201207256)
Supplement: Supplementary file 1 [file anie0051-12613-SD1.pdf]

Angewandte Chemie
Eine Zeitschrift der Gesellschaft Deutscher Chemiker

Supporting Information
© Wiley-VCH 2012
69451 Weinheim, Germany

Dissolution of Platinum: Limits for the Deployment of Electrochemical Energy Conversion?**

Angel A. Topalov, Ioannis Katsounaros, Michael Auinger, Serhiy Cherevko, Josef C. Meier, Sebastian O. Klemm, and Karl J. J. Mayrhofer**

anie_201207256_sm_miscellaneous_information.pdf

Experimental Section

The measurements were performed using a SFC coupled with an ICP-MS (NexION 300X, Perkin Elmer) (see supporting figure SF1). A polycrystalline Pt foil (99.99% MaTecK) was ground, polished, annealed and afterwards used as the working electrode. The area of the electrode exposed to the electrolyte was 1.1 mm^2. A carbon fiber was used as the counter electrode and a µ-Ag/AgCl as the reference electrode. The electrolyte was freshly prepared from perchloric acid (Suprapur® Merck) and ultrapure water (18.2 MΩ, PureLab Plus system, Elga) and continuously de-aerated by an Ar stream (Air Liquide, 5.0). The rate of the electrolyte flow through the cell was typically adjusted to 3.2 µL s^{-1}. The current/potential response was recorded by a Gamry Ref600 potentiostat. The Pt concentration in the electrolyte stream was measured by the ICP-MS following the intensities of ^{194}Pt and ^{195}Pt isotopes with respect to a 7.5 µg L^{-1} internal standard of ^{187}Re. Calibration of the ICP-MS response was performed before each measurement. Normalization of measured currents and dissolution rates was performed using the geometric contact area between the working electrode and the flow cell. The real surface area (roughness factor of *ca.* 1.3) was not taken into account since it might change during the experiments.

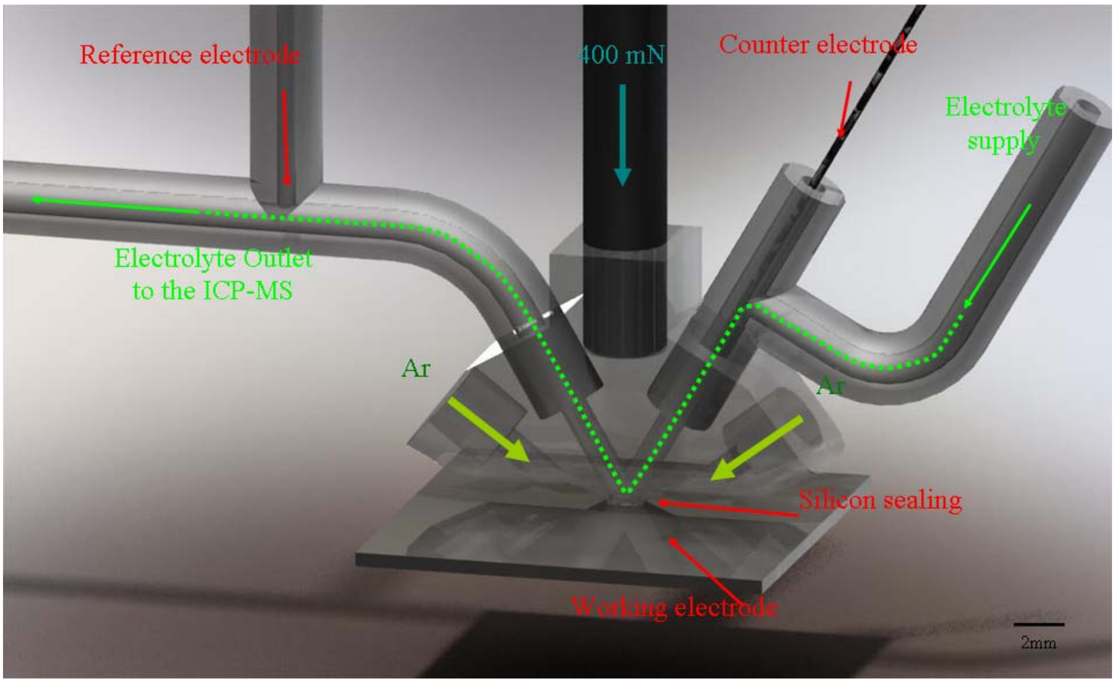

Supporting Figure 1. CAD model of the scanning flow cell setup. The green dotted line indicates the electrolyte flow (3.2 µL s^{-1}) through the channels of 1 mm internal diameter. The electrolyte flow rate is controlled by a peristaltic pump MP^2. The carbon counter electrode is placed in the inlet channel whereas the micro-reference electrode in a side-compartment connected to the outlet channel to avoid chloride contamination. A silicon sealing prevents the electrolyte from leaking over the entire sample and thus defines the area of the working electrode in contact with the electrolyte.
